# Management of Pediatric Tumors With Vascular Extension

**DOI:** 10.3389/fped.2021.753232

**Published:** 2022-01-04

**Authors:** Mayara Caroline Amorim Fanelli, José Cícero Stocco Guilhen, Alexandre Alberto Barros Duarte, Fernanda Kelly Marques de Souza, Monica dos Santos Cypriano, Eliana Maria Monteiro Caran, Henrique Manoel Lederman, Maria Teresa de Seixas Alves, Simone de Campos Vieira Abib

**Affiliations:** ^1^Department of Pediatric Surgery, Pediatric Oncology Institute, Grupo de Apoio ao Adolescente e à Criança com Câncer (GRAACC), São Paulo, Brazil; ^2^Department of Cardiovascular Surgery, Federal University of São Paulo, São Paulo, Brazil; ^3^Pediatric Oncology Institute, Grupo de Apoio ao Adolescente e à Criança com Câncer (GRAACC), São Paulo, Brazil; ^4^Department of Pediatric Oncology, Pediatric Oncology Institute, Grupo de Apoio ao Adolescente e à Criança com Câncer (GRAACC), São Paulo, Brazil; ^5^Department of Pediatric Radiology, Pediatric Oncology Institute, Grupo de Apoio ao Adolescente e à Criança com Câncer (GRAACC), São Paulo, Brazil; ^6^Department of Pediatric Pathology, Federal University of São Paulo, São Paulo, Brazil; ^7^Department of Pediatric Surgery, Federal University of São Paulo, São Paulo, Brazil

**Keywords:** pediatric tumors, vascular extension, Wilms tumor, cavotomy, cavectomy, hepatoblastoma, adrenocortical carcinoma, surgery

## Abstract

**Background:** Pediatric tumors can present with vascular extension to the inferior vena cava and right atrium, which impacts the surgical strategy and can be challenging during surgical treatment. Wilms tumor (WT) is the most common retroperitoneal tumor that can present with vascular extension, but also adrenal tumors, clear cell tumors from the kidney, and hepatoblastomas can present with this situation. Surgical aims include obtaining complete tumor resection without risk for patients, to avoid severe bleeding, cardiac arrest, and embolization, and to avoid cardiac bypass if possible.

**Objective:** To describe and discuss the surgical strategies to deal with pediatric tumors with vascular extension and propose a protocol.

**Method:** Retrospectivly review the experience of treating patients with vascular extension in a single institution, describing different scenarios and a decision making fluxogram based on the preoperative evaluation regarding the surgical techniques and the need for cardiac bypass that are adequate for each situation. Image studies are important to guide the surgical strategy. Depending on the quality of image available, computerized tomography (CT) or magnetic resonance imaging (MRI) can be enough to give the information needed for surgical decisions. Ultrasonography (US) with Doppler is helpful to confirm diagnosis and describes factors to guide the adequate surgical strategy, like the upper level extension and presence or absence of blood flow around the thrombus. Neoadjuvant chemotherapy is indicated in most cases, in order to reduce the upper level of extension (and avoid the need for cardiac bypass) and to lower the risk of embolization. The approach is based on the upper level of the thrombus and can include cavotomy or cavectomy, sometimes with cardiac bypass and cardiac arrest with hypothermia, when the thrombus reaches the diaphragmatic level or above. Pathology analysis of the thrombus can guide staging and the need for radiotherapy postoperatively.

**Results:** A decision making fluxogram protocol is presented focusing on the surgical treatment of such condition.

**Conclusion:** Surgery strategy is highly impacted by the presence of vascular extension in pediatric tumors. Surgeons should be aware of potential complications and how to prevent them. Such cases should be treated in reference centers.

## Introduction

Vascular extension to the inferior vena cava and right atrium can be challenging for surgical treatment of pediatric tumors. Wilms tumor (WT) is the most common retroperitoneal tumor with vascular extension (1), but other diagnoses can be found, like adrenal tumors, clear cell tumor from the kidney, and hepatoblastoma. Although uncommon, some clinical signs can suggest vascular extension, for example, varicocele, hepatomegaly, and collateral circulation (2, 3). Symptoms related to cardiac failure and dyspnea may occur in some cases.

The management requires a detailed preoperative evaluation. Neoadjuvant chemotherapy is indicated in most cases, in order to reduce the upper level of extension, to lower the risk of embolization, and mainly to avoid cardiac bypass (CBP) (4).

In cases of WT, despite the differences on the initial approach between the cooperative groups Societé Internationale d'Oncologie Pediatrique (SIOP) in Europe and Children's Oncology Group (COG) in North America, both agree with the use of preoperative chemotherapy in cases with vascular extension, in an attempt to reduce the level of the extension, minimizing the surgical risks and the need for cardiac bypass (CBP) (5–9). The number of preoperative chemotherapy cycles depends on the tumor histology and its thrombus response. There is no formal guidance regarding this issue.

Therefore, there are some factors that should be mentioned along with the preoperative evaluation (1):

- Tumor histology and stage at presentation
- Response to chemotherapy
- Confirmation of the vascular extension and its level
- Presence of blood flow around the thrombus
- Risk of tumor embolization

The extension of the thrombus can be classified in 3 levels, according to the hepatic veins:

- Level 1: infrahepatic thrombus
- Level 2: retrohepatic thrombus (subdivided in infradiaphagmatic and diaphragmatic)
- Level 3: supra hepatic or atrial thrombus

Image studies are of the essence to guide the surgical strategy. Depending on the quality of image available, either computerized tomography (CT) or magnetic resonance imaging (MRI) can be enough, but if there is doubt, ultrasonography (US) with Doppler should be performed to confirm the factors previously mentioned, like the upper level extension and blood flow (3, 6). Transesophageal echocardiogram under anesthesia also can be useful to confirm the thrombus presence, right before the procedure and also after resection (10). However, its approach is less sensible to retro hepatic and lower segments of inferior vena cava (IVC) (1).

Although there is controversy in the literature, preoperative anticoagulation should be indicated only when there is high risk of embolization, symptoms, and clinical impairment.

We aim to describe lessons learned from dealing with such cases and suggest a protocol to manage it.

## Methods

In our institution, in the past 24 years, we have registered 40 patients whose tumors presented with vascular extension and were evaluated for surgery. The diagnoses included WT, hepatoblastoma, adrenocortical carcinoma (ACT), and primitive neuroectodermal tumor (PNET) of the kidney.

Vascular extension was identified mainly after CT, but also US with Doppler was used in some cases to confirm the upper level and blood flow status. In one case, anticoagulation was performed, in order to prevent embolization, due to a pedicled tumor through the tricuspid valve and clinical symptoms. Anticoagulation was not indicated in other patients of the series.

The surgical outcomes will be presented below.

## Surgical Technique

The surgical approach is based on the upper level of the thrombus and the presence or absence of blood flow around it. Usually, a midline laparotomy incision is recommended, which can be extended to the chest, if it is necessary, but associated sternotomy and transverse laparotomy provide good exposure as well. Since some patients may need CPB, it would be advisable to minimize muscular section, due to the need of anticoagulation. Most techniques are derived from the liver transplant surgical approach, which allows a good exposure and safe vascular control along the procedure (10).

**Figure 1:**
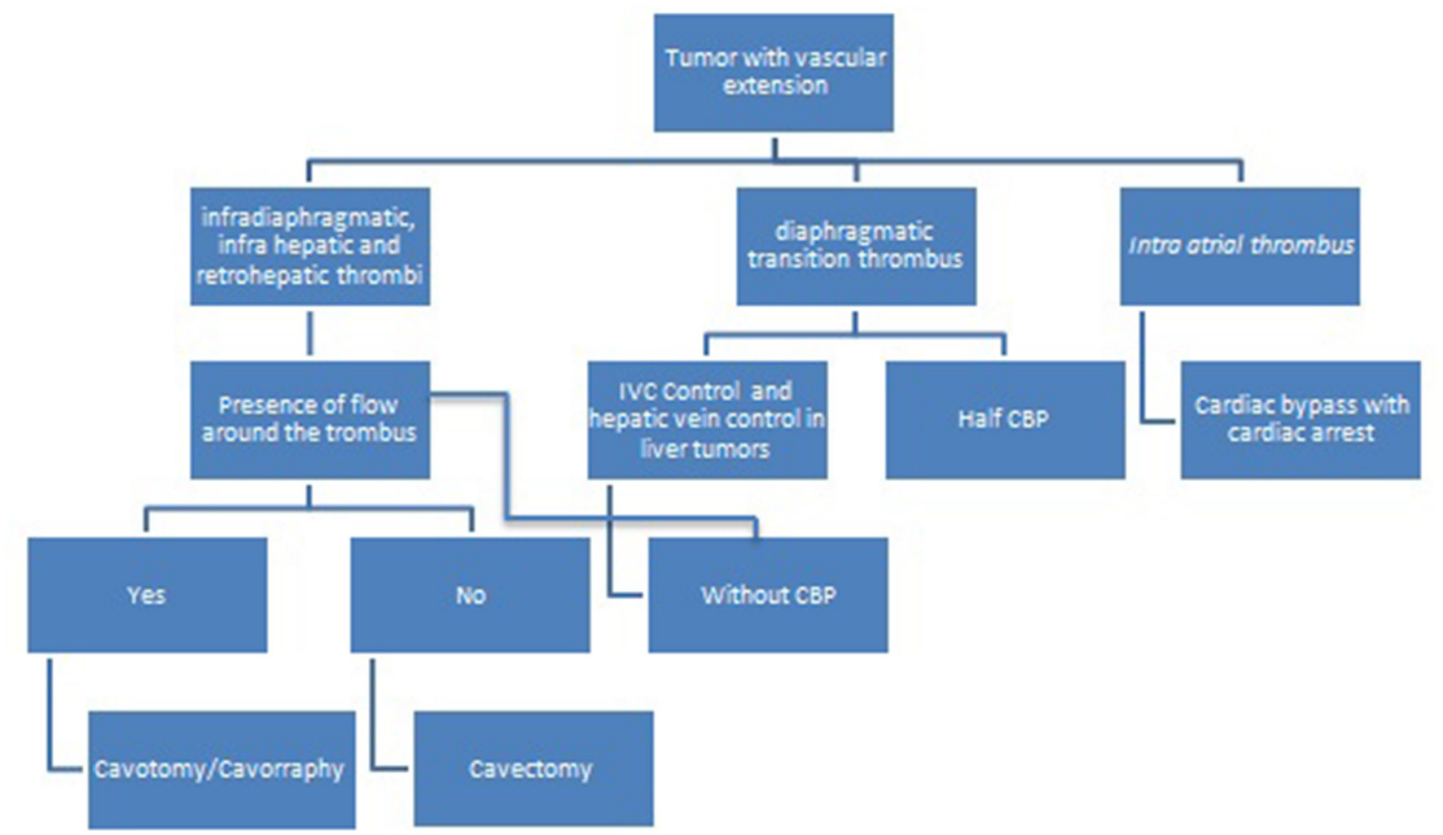
Decision-making fluxogram for surgical treatment of pediatric tumors with vascular extension.

Based on preoperative information about thrombus level and blood flow around it, the following protocol was designed (2) (Figure 1).

### Infradiaphragmatic Thrombus

For infradiaphragmatic thrombus, either infra or retro hepatic below the diaphragmatic transition, there is no need for CBP, heparinization, or cardiac arrest. IVC vascular control below the liver and distally, along with renal vein vascular is enough to achieve complete tumor resection, either by cavotomy or cavectomy. In this scenario, blood loss is minimal and the ischemic time to the kidney with this technique is uneventful (4).

If there is blood flow around the thrombus, cavotomy is performed and the thrombus can be easily removed since there is no invasion to the vessel wall.

In the case of blood flow absence around the thrombus, it is firmly adherent and invades the vessel wall, thus cavectomy is indicated and no anticoagulation is needed.

Contralateral renal vein ligation is justified and safe due to the development of collaterals venous of the azygo's system while the tumor grows, which is responsible to keep adequate venous return to the contralateral kidney or adrenal (2, 4, 5). For this reason, there is no need for IVC prosthesis replacement. In addition, the cavectomy might reduce the need for post-operative radiotherapy, due to the complete resection of the tumor (11).

### Diaphragmatic Thrombus

For tumors with thrombus in the diaphragmatic transition, it is advisable to have a cardiac surgery team backup, for the decision of CBP may be intraoperative. If CBP is not needed, the already described approach for infradiaphragmatic thrombus should be performed. In our initial cases with this scenario, we indicated resection with CBP, but we learned that this can be avoided in many cases.

If the vascular control at the upper level is not secure, a small sternotomy should be made in order to achieve IVC control and a “half” CBP without cardiac arrest and without hypothermia is indicated. In this case, the patient receives heparinization (2 mg/Kg), blood is recovered from the surgical field, is filtered and warmed, and cannulation of the aorta or iliac artery is made, in order to provide rapid fluid replacement without CBP.

Either cavotomy or cavectomy can be performed, depending on the presence or absence of blood flow around the thrombus, as described above, with or without CBP.

### Intracardiac Thrombus

Finally, for tumors with intracardiac extension, CPB is always needed, associated with cardiac arrest and hypothermia (3, 12). This strategy provides a surgical field without blood, and prevents cardiac arrest due to severe bleeding and thromboembolism during IVC manipulation. In addition, the presence of intra-atrial tumor can obstruct the venous return especially in smaller children. Initially, we used profound hypothermia in all cases (18°C), but if cardiac arrest takes <30 min, moderate hypothermia (20–22°C) can be made. For cases in which there are longer periods of cardiac arrest (more than 40 min), moderate hypothermia of 20–22°C plus selective cerebral perfusion can be made. It is well-known how harmful hypothermia is for children, so it is advisable to reduce hypothermia times.

## Results

There were 40 patients identified with vascular extension at the initial investigation, six of them were excluded due to lack of data. From 34 patients included, 21 were WT, one hepatoblastoma, 11 adrenocortical carcinomas (ACT), and one PNET of the kidney.

**Figure 2:**
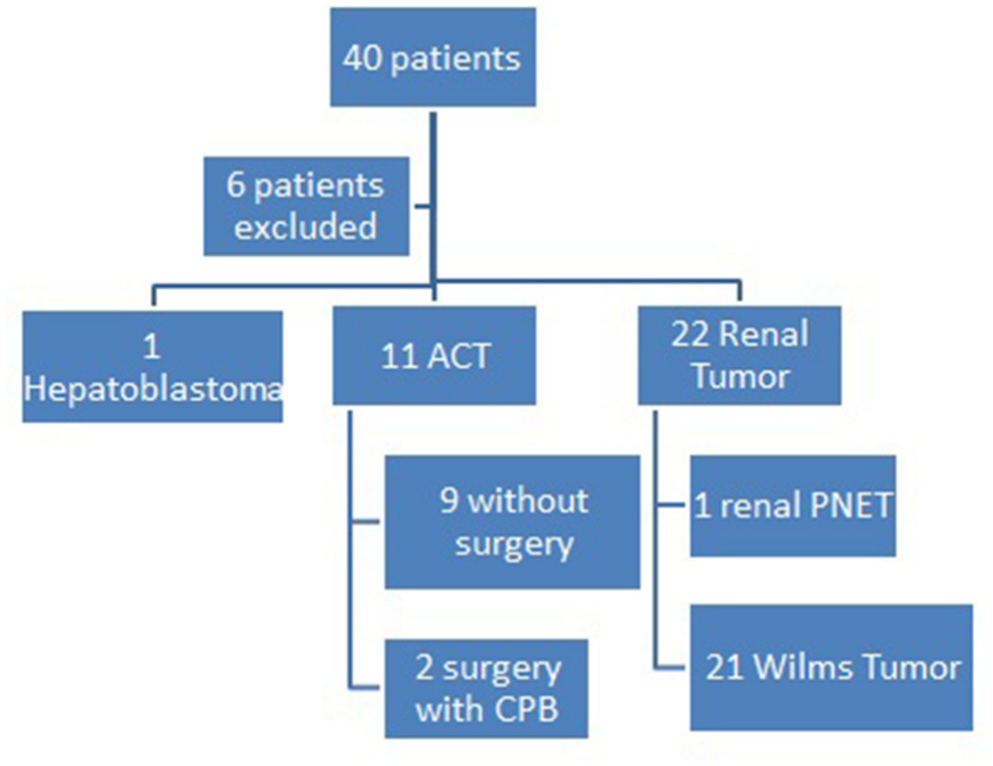
Pediatric Oncology Institute - GRAACC casuistic on pediatric tumors with vascular extension.

Only two patients with ACT were submitted to the surgical approach due to clinical conditions. In both of them, there was a thrombus reaching the atrium and they were submitted to cavotomy with CPB. One of them died due to bypass system obstruction with thrombus. The other one had neurological postoperative complications related to CBP and died 4 years after the procedure of progression of disease (Figure 2).

Renal and hepatic tumors were first treated with preoperative chemotherapy. There was complete thrombus regression in six patients, five of them with WT and one of them with renal PNET, with no need for the vascular approach.

Partial regression of the thrombus occurred in 17 patients, who needed vascular management. Among them, we performed eight cavotomies and five cavectomies. Four patients still presented atrial thrombus after chemotherapy and needed CPB.

We achieved complete remission in 13 patients. Five patients lost follow up after ending adjuvant chemotherapy and radiotherapy, five patients had died due to complications not related to surgery, and one patient is still under treatment.

## Discussion

Surgical management changes in the presence of vascular extension and the present paper proposes a fluxogram to guide the surgical treatment, that can be used both for retroperitoneal and intraperitoneal pediatric tumors with vascular extension (Figure 1). Some details are further described according to tumor characteristics.

In renal cell carcinoma, vascular extension occurs in 10% of patients (13) and it is an independent predictor of survivorship. The curative potential is determined by the complete surgical resection, including the tumor and the thrombus (10), as well as in ACT. In WT, survival does not change due to thrombus presence and preoperative chemotherapy can modify the thrombus level and avoid the need for cardiac bypass. For this reason, different protocols indicate preoperative chemotherapy in such cases (8, 9, 14, 15). Even with careful preoperative workup, it is advisable that surgeons palpate both the IVC and renal vein, in order to avoid the transection of an unidentified vascular extension during the surgical approach because it can upstage the tumor to stage III (16). In WT, upstaging to stage III will depend on lymph node status, complete resection of the thrombus (or not), and the presence or absence of viable cells within the thrombus. Stage III requires postoperative radiotherapy.

Some authors advise radiotherapy to treat the thrombus instead of surgical resection (5), but we think that depending on the presence or absence of viable cells within the thrombus, radiotherapy may be avoided if complete surgical resection is achieved.

In ACT, the IVC extension occurs in 20% of the cases. It is more frequent at the right side (13) and is related to very poor prognosis. Entire thrombus resection with the tumor is essential to the curative treatment, although it can be challenging. In our experience, ACT thrombi are usually friable, unlikely to shrink with preoperative chemo, and impacts staging and the oncological prognosis. Because of that, surgical complications are more frequent. On the other hand, comparatively, thrombi from WT tend to be firm, may regress and shrink after chemotherapy, and surgical complications are rare, in the sense that its presence does not change the stage or the oncological prognosis (17).

In hepatoblastomas, vascular involvement adds risk to the stratification based on the European based International Childhood Liver Tumors Strategy Group (SIOPEL) Pre Treatment Extent of disease (PRETEXT), as shown in Pediatric Hepatic International Tumor Trial (PHITT). Vascular extension is one of the Annotation Factors and they are considered positive when there is: tumor involvement of all 3 hepatic veins or retrohepatic vena cava and/or tumor thrombus in any 1 or more of the main hepatic veins; tumor involvement of the portal bifurcation, both right and left portal veins; and/or tumor thrombus in either the left or right portal, and others related to the involvement to other organs (18). The vascular extension can occur through the hepatic veins until the atrium, but the development of vascular reconstruction techniques has increased the resection rates over time (19). In our reported case of hepatoblastoma with vascular extension to the atrium, first were performed the thrombus resection by sternotomy with cardiac bypass and cardiac arrest. After the cardiac bypass ceased, the liver mass was resected by laparotomy without complications and the patient was discharged home after 6 days. The tumor relapsed and the patient was submitted to liver transplantation. Unfortunately, he died due to post-transplant complications.

Pediatric tumors with vascular extension demand careful multidisciplinary discussions and preoperative preparation. They should be referred to high complexity surgical centers.

## Conclusion

In conclusion, surgery strategy is highly impacted by the presence of vascular extension in pediatric tumors. Despite the protocols, the best approach is determined case by case, considering the preoperative evaluation. The risks are justified because the tumor resection is of essence for treatment. Surgeons should be aware of potential complications and how to prevent them. Such cases should be treated in reference centers, always remembering the importance of a multimodal assistance, including the pediatric oncologist, pediatric radiologists, pathologists, and pediatric and cardiac surgeons.

## Data Availability Statement

The original contributions presented in the study are included in the article/supplementary material, further inquiries can be directed to the corresponding author.

## Ethics Statement

The studies involving human participants were reviewed and approved by Comitê de ética e Pesquisa UNIFESP - n° 1015/2019. Written informed consent was not required for this study, in accordance with the local legislation and institutional requirements.

## Author Contributions

All authors listed have made a substantial, direct, and intellectual contribution to the work and approved it for publication.

## Conflict of Interest

The authors declare that the research was conducted in the absence of any commercial or financial relationships that could be construed as a potential conflict of interest.

## Publisher's Note

All claims expressed in this article are solely those of the authors and do not necessarily represent those of their affiliated organizations, or those of the publisher, the editors and the reviewers. Any product that may be evaluated in this article, or claim that may be made by its manufacturer, is not guaranteed or endorsed by the publisher.
